# Neural correlates of perceptual separation-induced enhancement of prepulse inhibition of startle in humans

**DOI:** 10.1038/s41598-017-18793-x

**Published:** 2018-01-11

**Authors:** Ming Lei, Changxin Zhang, Liang Li

**Affiliations:** 1 School of Psychological and Cognitive Sciences, Beijing Key Laboratory of Behavioral and Mental Health, Peking University, Beijing, 100080 China; 2 Department of Health Industry Management, Beijing International Studies University, Beijing, 100024 China; 3 Faculty of Education, East China Normal University, Shanghai, 200062 China; 4 Key Laboratory on Machine Perception (Ministry of Education), Speech and Hearing Research Center, Peking University, Beijing, 100871 China; 5 Beijing Institute for Brain Disorders, Beijing, 100069 China

## Abstract

Prepulse inhibition (PPI) is the suppression of the startle reflex when the intense startling stimulus is shortly preceded by a weaker non-startling stimulus (prepulse). In rats, the auditory precedence-effect-induced perceived spatial separation between the fear-conditioned prepulse and a noise masker facilitates selective attention to the prepulse and enhances PPI. However, whether the perceptual separation between the prepulse and a noise masker can also enhance PPI in humans remains unclear. Also, the relationship between the PPI enhancement and the change in early cortical representations of prepulse signals is unclear. This study for the first time reveals that in a sound-attenuated laboratory environment, relative to the listening condition with perceptual co-location between the prepulse stimulus and a noise-masking stimulus, the perceptual separation between the two stimuli significantly enhances the group-mean PPI. More importantly, the early cortical responses (N1/P2 complex) to the prepulse stimulus are also enhanced by the perceptual separation in most listeners, and the perceptual-separation-induced enhancement of the N1 component is positively correlated with the perceptual-separation-induced PPI enhancement. Thus, the perceptual separation enhances PPI through facilitating selective attention to the prepulse, leading to an enhancement of the early cortical representation of the prepulse signal in temporal auditory cortical fields.

## Introduction

The startle reflex is the whole-body reflexive response to sudden and intense sensory stimuli, which disrupts cognitive/behavioral performances^1^. Prepulse inhibition (PPI) is the normal reduction of the startle reflex when a weaker sensory stimulus (the prepulse) shortly precedes the startling stimulus^2,3^. The “protection-of-processing” theory proposed by Graham (1975) suggests that receiving a prepulse simultaneously triggers both the sensory processing for the prepulse and the gating mechanism dampening the disrupting influence from startle^4^. Since the consequences of PPI include the reduction of behavioral responses to disruptive stimuli by regulating the motor/premotor system, PPI has been generally recognized as an operational measure of sensorimotor gating. The level of PPI has also been considered as a measure of the salience of the prepulse stimulus in both rodents and humans^5^.

Although PPI is thought to be automatic, previous studies have shown that PPI can be top-down modulated by attention and emotion^5^. In humans, attention can modulate the magnitude of PPI^6,7^. In an active PPI paradigm (when participants are asked to attend to the prepulse), PPI is greater when the prepulse is attended than when ignored^8,9^. In rats, selective attention to the prepulse also enhances PPI: PPI is larger when the prepulse is emotionally salient than when it is emotionally neutral^10^. Particularly, when a prepulse becomes fear conditioned in rats, it draws more attention and elicits larger PPI^11–13^. More interestingly, in rats, when a noise masker is presented, the auditory precedence-effect-induced perceptual separation between the fear-conditioned prepulse and the noise masker further enhances PPI^14–18^.

What is the precedence effect? In a reverberant environment, listeners receive not only the direct wave from a sound source but also numerous time-delayed reflections of the source. If the time delays between the arrival of the direct wave and each of the reflected waves are sufficiently short (e.g., 1–10 ms or more, depending on the nature of the stimulus), due to the perceptual capture of the attributes of the delayed and correlated reflections by the direct wave from the source^19^, listeners typically perceive a single “fused” image of the source located at or near the original site of the source. This phenomenon has been generally known as the precedence effect^20,21^. The minimum delay allowing a listener to perceive the lagging sound as a discrete echo is called the echo threshold^20^, indicating the perceptual fusion tendency (a large echo threshold suggests a large perceptual fusion tendency). The perceptual fusion will be broken at delays larger than the echo threshold. As the most important acoustic stimuli for human communication, speech sounds contain distinct patterns of periodicities and transients, and cause larger echo thresholds (over 14 ms) than other types of sounds such as noise bursts^21,22^. Although the precedence effect for speech sounds can occur at any lead-lag delays shorter than the echo threshold, it has been confirmed that when a 3-ms delay is introduced beteween the leading speech sound and the correlated lagging speech sound (which simulates the reflection of the leading speech sound), not only the leading and lagging speech sounds are well fused perceptually, but also a single speech-sound image is perceived as coming from the location of the leading speech sound^21–24^. Thus, the delay of 3 ms was also used in this study.

In humans, when both a target speech sound and a masker sound (noise or speech) are presented by each of the two spatially separated loudspeakers with an inter-loudspeaker delay of 3 ms, the target and the masker are perceived as either from the same loudspeaker when the target and masker share the same leading loudspeaker (the perceived co-location condition), or from different loudspeakers when the leading loudspeaker for the target is different from that for the masker (the perceived separation condition). Importantly, recognition of the target speech under the condition of perceived target-masker separation (when the leading loudspeaker for the target is different from that of the masker) is significantly better than that under the condition of perceived co-location^22–24^, even though neither the masker energy at each ear nor the sound-image compactness/diffusiveness is changed^23^. The enhancement of recognition is caused by the promotion of listener’s selective spatial attention to the target signal, and is associated with activation of some spatial attention-related brain regions, such as the superior parietal lobule^24^.

In rats, when the conditioned prepulse and a noise masker are perceived spatially separated, selective attention to the conditioned prepulse signal is further facilitated, leading to that PPI is further enhanced^14–18^. Moreover, in the rodent model of schizophrenia induced by social isolation rearing, perceptual separation-induced PPI enhancement completely disappears^14,15,18^.

Although people with schizophrenia exhibit deficits in PPI^25^, only the impairment of the attentional modulation of PPI (but not impairment of the baseline PPI) is significantly correlated with the symptom severity of schizophrenia^26,27^. To establish a new paradigm for examining attentional modulation of PPI in humans, it is of interest and importance to know whether the perceptual separation between the prepulse stimulus and a masker can enhance PPI in healthy humans. The first purpose of this study was to investigate whether perceptual separation between the prepulse stimulus and the noise masker can affect PPI in healthy young human adults.

The N1/P2 ERP complex, a group of components of the early cortical auditory-evoked potentials, can be reliably elicited by various acoustic stimuli (e.g. single syllables, noise burst, pure tones), even when a noise or a speech masker is co-represented^28,29^. Zhang *et al*. (2014) have recently reported that a target syllable /bi/ induces steady N1/P2 complex when the target is co-presented with a noise masker or a speech masker. More importantly, perceived separation between the target and the masker facilitates selective attention to the target and further enhances the N1/P2 complex when the speech masker is actively listened^29^.

Note that although the N1 component and the P2 component are elicited simultaneously, these two components are generated by different brain regions^30,31^. The N1 component may be located in the temporal auditory cortical fields, including both the Heschl’s gyrus and the superior temporal polysensory area (STP)^31,32^. Moreover, Woods *et al*. (1993) have reported that lesions of the inferior parietal lobe (IPL), which plays a role in auditory spatial attention, reduced the amplitude of N1, but not P2^33^. Differently, the P2 component may reflect, at least partly, acoustically driven outputs from the mesencephalitic reticular activating system that also responds to inputs from multiple sensory modalities^34^. A recent ERP study has shown that N1 is influenced by stimulus-driven attention to the prepulse, while P2 is influenced by goal-directed attention to the prepulse^7^. Thus, the second purpose of this study was to examine whether the perceptual separation between the prepulse stimulus and the masking noise affects the two ERP components (N1 and P2) to the prepulse stimulus in different manners.

In a sound-attenuated laboratory listening environment^21,22,29,35,36^, this study was to examine whether the perceptual separation between the prepulse and a noise masker can modulate PPI and/or affect ERPs to the prepulse stimulus in human adults with normal hearing.

## Results

### PPI Enhancement Induced by Perceptual Separation

In Experiment 1, to maintain participants’ attention to acoustic stimuli, participants were asked to report the number of the prepulse in each session. All the 18 participants reported 24 ± 2 in each session, showing that their attention to acoustic stimuli was well maintained.

**Figure 1:**
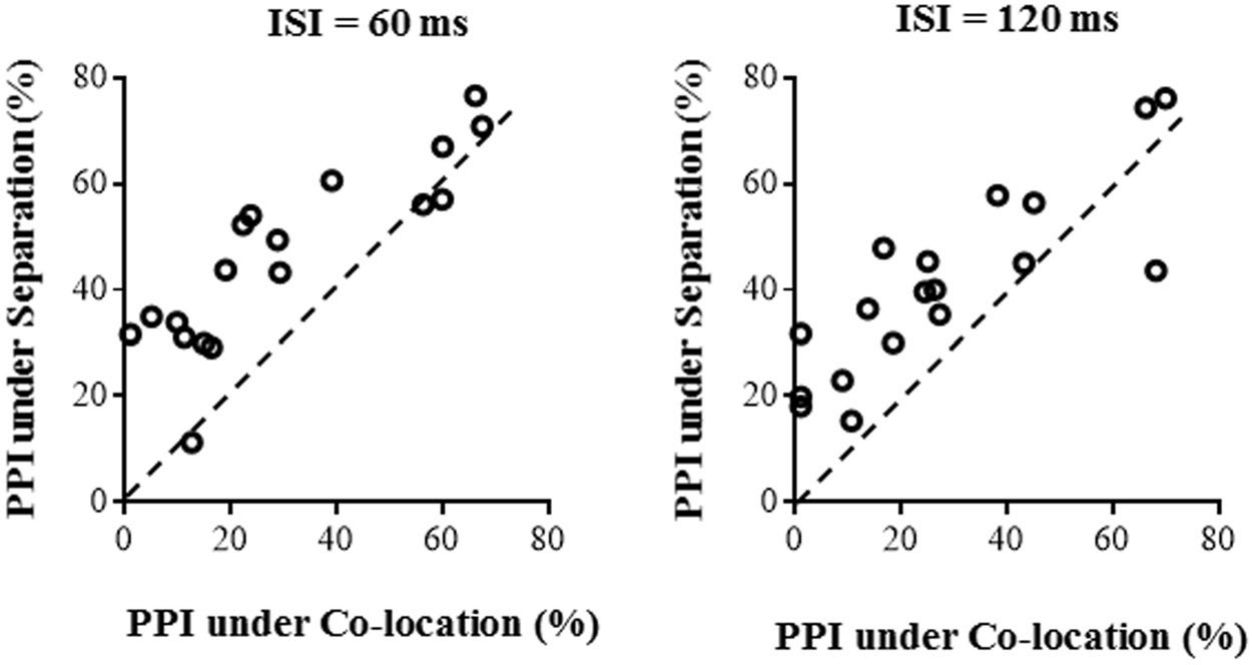
PPI values under either the perceptual co-location condition or the perceptual separation condition for individual participants in Experiment 1, when the inter-stimulus interval (ISI) between the prepulse and the startling sound was either 60 or 120 ms.

Figure 1 shows the PPI values of the 18 individual participants under the co-location condition (values along the abscissa) and those under the separation condition (values alone the ordinate). As shown in Fig. 1, PPI values under the separation condition were larger than the PPI values under the co-location condition for most participants when the ISI was either 60 or 120 ms.

**Figure 2:**
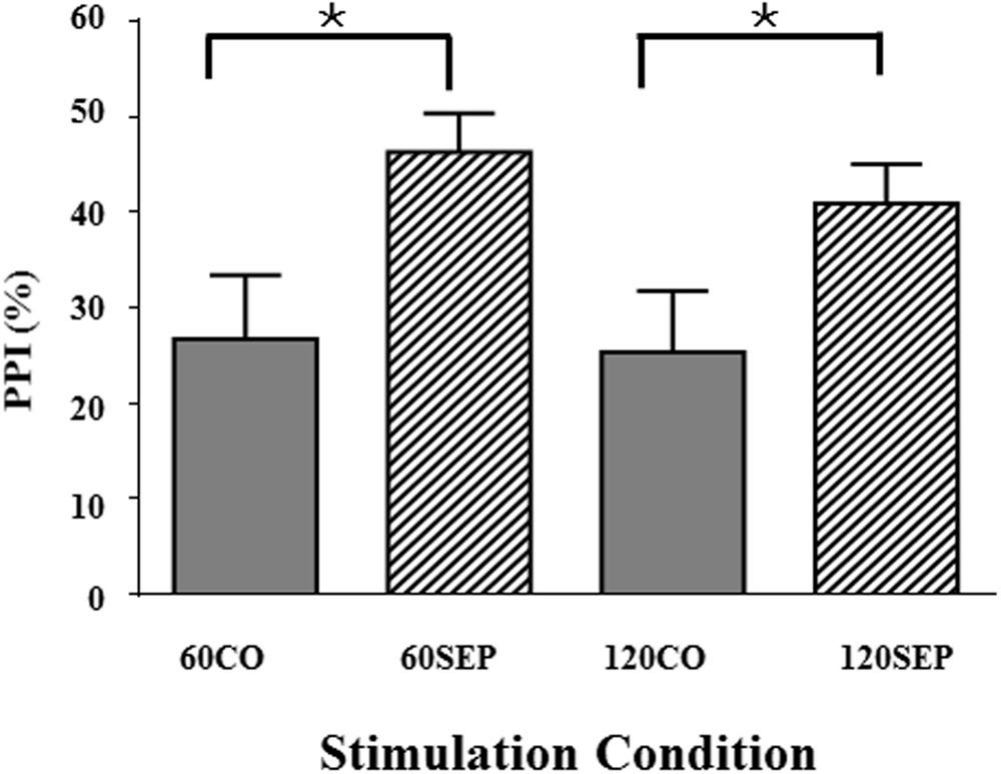
Comparisons of the PPI magnitude between the perceptual co-location condition and the perceptual separation condition when the ISI was either 60 or 120 ms. 60CO, perceptual co-location condition with the ISI of 60 ms; 60SEP, perceptual separation condition with the ISI of 60 ms; 120CO, perceptual co-location condition with the ISI of 120 ms; 120SEP, perceptual separation condition with the ISI of 120 ms. *p < 0.05.

To statistically examine the effects of perceptual separation and ISI (Fig. 2), a 2 (separation type: separation, co-location) by 2 (ISI: 60 ms, 120 ms) within-subject repeated-measures ANOVA revealed that only the main effect of separation type was significant (*F*_1,17_ = 21.65, *p* = 0.008). Post hoc tests showed that PPI was enhanced by perceived spatial separation, regardless of whether the ISI was either 60 or 120 ms. Thus, when the prepulse and the noise masker were perceptually separated, the PPI magnitude was higher than that when the prepulse and the noise masker were perceptually co-located.

### Correlation between PPI Enhancement and ERP Enhancement

**Figure 3:**
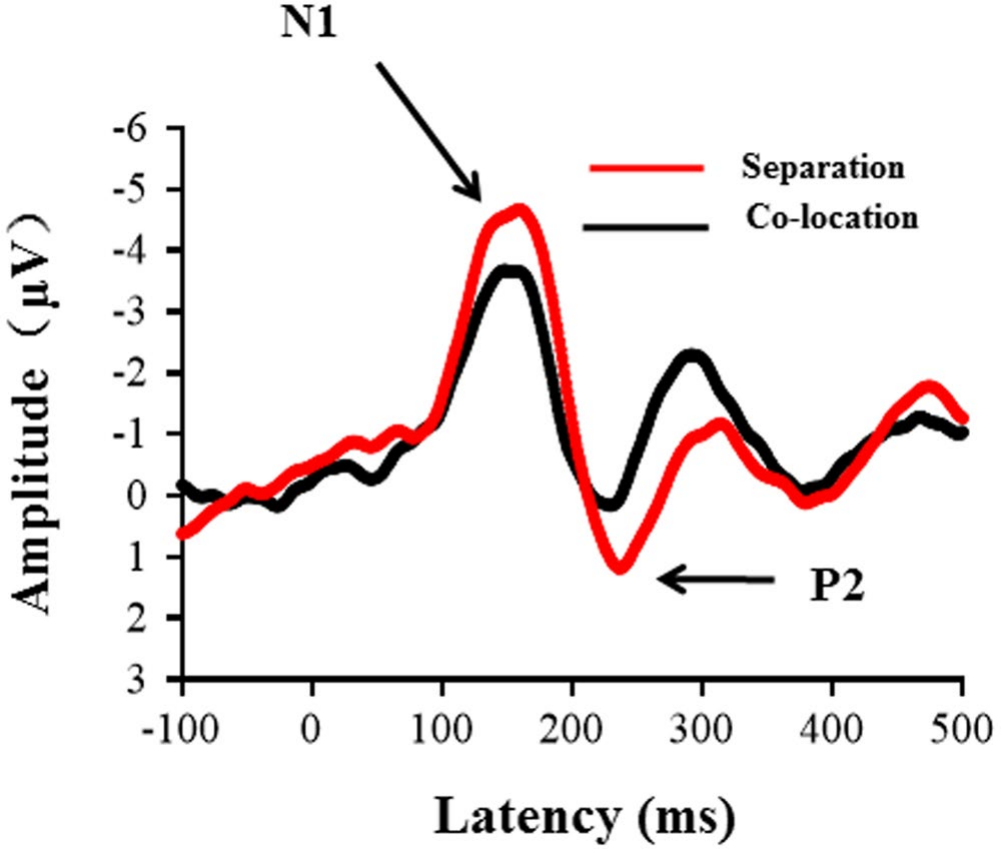
Group-mean ERP waveforms to the prepulse stimulus, recorded from the electrode site Cz across 20 participants under either the perceptual co-location condition or the perceptual separation condition. The prepulse stimulus evoked a larger N1/P2 complex when the prepulse and the masker were perceptually separated relative to when the perceptual co-location condition.

Grand-mean ERP waveforms to the target sound (i.e., the prepulse stimulus as used in Experiment 1) recorded from the electrode site Cz across participants are shown in Fig. 3. As Fig. 3 shown, the target stimulus evoked a larger N1/P2 complex when the masker and the target were perceptually separated than when the masker and the target were perceptually co-located.

**Figure 4:**
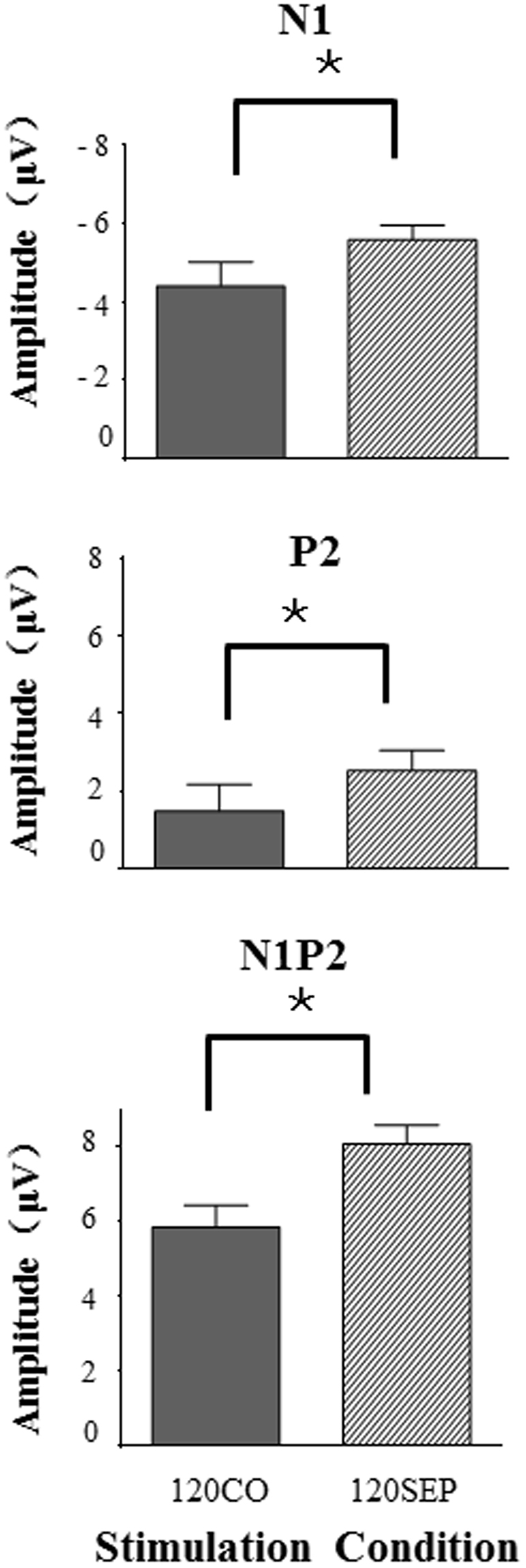
Comparisons of the group-mean N1, P2, and N1/P2 complex amplitudes to the prepulse stimulus recorded at the electrode site Cz between the perceptual co-location condition and the perceptual separation condition.

The average values of N1 peak, P2 peak, and N1/P2 peak-to-peak amplitudes to the target under either the co-location condition or the separation condition were displayed in Fig. 4. As shown in Fig. 4, relative to the co-location condition, perceptual separation between the target sound and the noise masker enhanced the values of N1 peak, P2 peak, and N1/P2 peak-to-peak amplitudes.

Separated paired *t* tests showed that the separation effect was significant for the values of N1 peak (*p* = 0.032), P2 peak (*p* = 0.026), and N1/P2 peak-to-peak amplitudes (*p* = 0.0002). However, the N1 and P2 latencies were not significantly affected by the listening condition (*p* = 0.408, *p* = 0.452, respectively).

**Figure 5:**
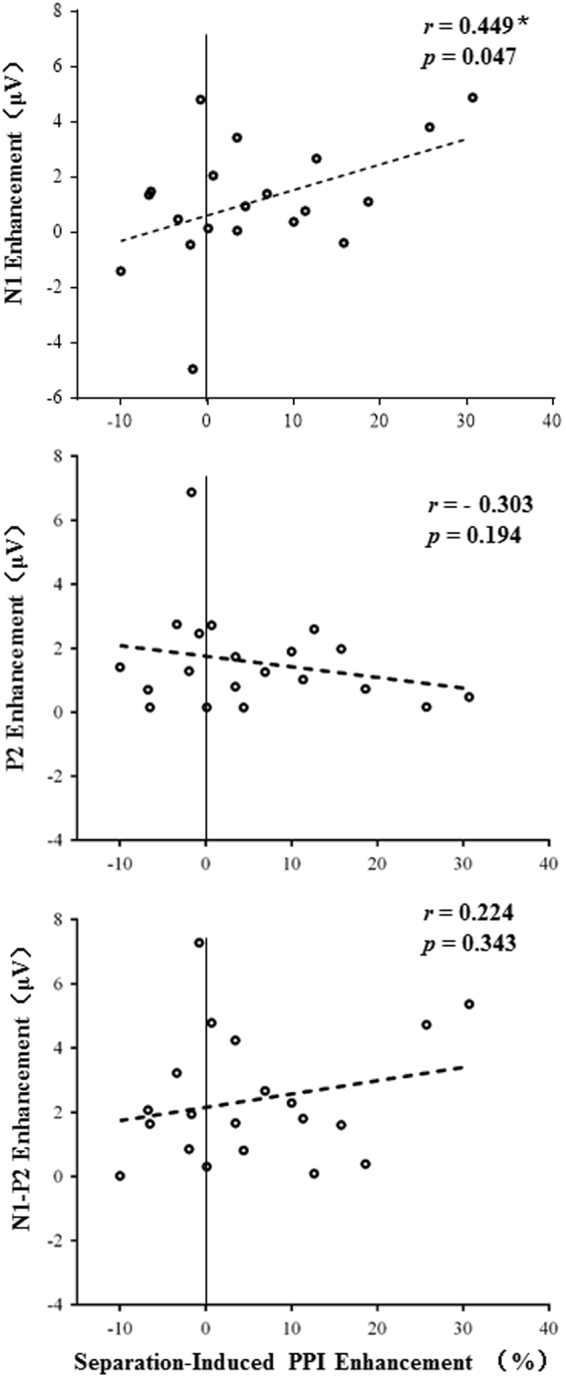
Correlations between the separation-induced enhancement of the early cortical representation (N1, P2, or N1-P2) of the prepulse stimulus and the separation-induced PPI enhancement. Only the enhancement of the N1 component, but not the P2 enhancement or the N1P2 complex, was significantly correlated with PPI enhancement.

Figure 5 shows the correlations between the PPI enhancement induced by perceptual separation and the separation-induced enhancements of the N1 peak amplitude, P2 peak amplitude, and N1/P2 peak-to-peak amplitude, respectively. Pearson correlation coefficients were calculated. Surprisingly, the separation-induced PPI enhancement was significantly correlated only with the N1-component enhancement (Adjusted *r* = 0.449, *p* = 0.047, top panel of Fig. 5), but not the P2-component enhancement or the N1/P2-complex enhancement (*p* = 0.194, *p* = 0.343, respectively, middle and bottom panels of Fig. 5).

## Discussion

In the present study, at the ISI (between the prepulse offset and the startle-stimulus onset) of either 60 or 120 ms, the PPI magnitude was larger in the majority of participants when the prepulse and the noise masker were perceptually separated than when the prepulse and the noise masker were perceptually co-located. The group-mean PPI magnitude under the perceptual separation condition was also significantly larger than that under the perceptual co-location condition. Note that shifts between the two listening condition do not alter either the signal (prepulse)-to-noise ratio or the compactness of sound images, thereby without affecting the peripheral processing. Thus, the separation-induced PPI enhancement was a consequence of the perceptual processing.

It has been known that the PPI level is determined by the salience and processing depth of the prepulse signal^5^. In humans, the precedence-effect-induced perceived spatial separation between target speech and masker facilitates selective spatial attention to the target signal stream and improves recognition of target speech^22,23^. The results of this study suggest that perceptual separation between the prepulse and the masker facilitates selective attention to the prepulse and then causes an enhancement of PPI.

The results of this study are also consistent with those of some previous studies using laboratory rats^12,13,15,17,18^. In these animal studies, when the prepulse became fear conditioned, it drew more attention and elicited larger PPI^12,13^. Furthermore, when the conditioned prepulse and a noise masker were perceived spatially separated, PPI was further enhanced by the perceptual separation^14–16^. Also, the perceptual separation-induced PPI enhancement shows both feature-based and location-based specificity^15,17,18^: Only the conditioned prepulse perceived coming from the conditioned location can elicit the enhancement of PPI^17^.

Previous studies of the attentional modulation of PPI have focused on feature-based attention to the prepulse, such as instructing participants to listen to tones with certain frequencies^26,27^. The perceptual separation-induced PPI modulation paradigm used in this study offers a new paradigm to examine the spatially attentional modulation of PPI. Clearly, further studies combining the feature-based and spatial location-based attentional modulation of PPI are needed to deeply investigate the complex mechanism underlying top-down modulation of PPI.

The results of Experiment 2 of this study showed that the perceptual separation of the target stimulus (the prepulse stimulus used in PPI testing) and the noise masker significantly enhanced the early cortical representation of the target signal (i.e., the N1/P2 complex to the prepulse stimulus). More importantly, across listeners, the N1-amplitude enhancement induced by the perceptual separation was positively correlated with the PPI enhancement induced by perceptual separation.

Previous psychoacoustic studies have shown that the perceptual separation between the target speech and a masker facilitates the listener’s selective attention to target speech and improves recognition of target speech^22,23^. Previous electrophysiological studies have also shown that at the active-listening condition but not the passive-listening condition, when listeners attend to a target syllable/bi/, perceptual separation of the target syllable from a speech masker enhances the N1/P2 complex to the syllable^29^. In the present study, since the participants were instructed to attend to the prepulse, an active-listening condition was introduced and the perceptual separation further facilitated selective attention to the prepulse, thereby enhancing the early cortical ERP components (N1/P2 complex). The results of this study are consistent with the previous reports^28,29,37^.

The results of this study suggests that when listeners attend to the target (the prepulse), moving the masker image away from the location of the target image facilitates selective attention to the target, thereby enhancing the early cortical representation of the prepulse. Moreover, since the N1 and P2 latencies are not affected by perceptual separation, perceptual separation mainly enhances the processing depth, but not the processing speed. Thus, we propose that because perceptual separation facilitates attention to the prepulse signal, it enhances the cortical representation of the prepulse signal and induces the PPI enhancement.

More interestingly, only the separation-induced N1 enhancement, but not the P2 enhancement, is positively correlated with the separation-induced PPI enhancement. Some studies have suggested that N1 and P2 are generated in different brain regions^30,31^. Generators of N1 are located in the temporal auditory cortical fields, including the Heschl’s gyrus and the superior temporal polysensory area (STP)^32^, but P2 is generated in the higher sensory cortex^34^. Moreover, N1, but not P2, is related to the function of the inferior parietal lobe (IPL), which plays a role in auditory spatial attention^33^. Thus, N1, compared to P1, is generated from the auditory cortical fields and more related to the auditory spatial attention. Annic *et al*. (2014) have shown that N1 is influenced by stimulus-driven attention to the prepulse, while P2 is influenced by goal-directed attention to the prepulse^7^. The results of this study suggest that perceptual separation facilitates spatial attention to the prepulse, and enhances the early cortical representation of the prepulse (N1 component) in the temporal auditory cortical fields, and then enhances PPI.

Since animal studies have also shown that the posterior parietal cortex (PPC) plays a role in mediating the perceptual separation-induced PPI enhancement^16^, further brain-imaging studies should be conducted to examine whether the human PPI enhancement induced by perceptual separation is based on the function of PPC^24^.

In conclusion, this study reveals that in human listeners, the auditory precedence-effect-induced perceptual separation between the prepulse stimulus and a noise masker, which does not affect the peripheral processing, significantly enhances both PPI and scalp ERPs to the prepulse stimulus. More importantly, the perceptual separation-induced PPI enhancement is positively correlated with the perceptual separation-induced enhancement of the N1 component of the early cortical representation of the prepulse signal, suggesting that the perceptual separation-induced PPI enhancement is caused by the enhancement of prepulse representation in the temporal auditory cortical fields when selective attention to the prepulse is facilitated by the perceptual separation.

## Methods

### Experiment 1: Effects of Perceptual Separation on PPI

#### Participants

Eighteen healthy adults (12 males and 6 females, mean age = 33.7 ± 8.7 years) participated in Experiment 1. All the participants were right-handed native Chinese speakers with normal (audiometric thresholds <25 dB HL between 250 and 8000 Hz) and bilaterally balanced hearing (interaural threshold differences at each of the frequencies did not exceed 10 dB). The participants were paid a modest stipend for their participation.

This study was conducted according to the principles expressed in the Declaration of Helsinki. The procedures of Experiment 1 and those of Experiment 2 of this study were approved by the Committee for Protecting Human and Animal Subjects of the Department of Psychology at Peking University. All participants gave written informed consent before their participation in this study.

#### Apparatus and stimuli

Each participant sat comfortably in a recliner chair in a sound-attenuated room^36^. Two 4-mm Ag/AgCl electrodes were positioned below and lateral to the right eye over the orbicularis oculi, with a ground electrode behind the right ear. Electrode resistances were <5 kΩ. The eyeblink component of the acoustic startle system was measured using a human EMG startle reflex system (EMG XEYE human startle reflex, Tianminghongyuan Instruments, Beijing, China)^38^. EMG activity was band-pass filtered (10–500 Hz) and amplified by 40,000. Electrical voltages were collected and sampled at a frequency of 1000 Hz for 450 ms (150 ms before and 300 ms after the startling stimulus onset). For a single trial, the maximum peak-to-peak amplitude of the startle response within the time window of 20–300 ms after the onset of startle stimulus was digitized and measured.

The prepulse was a burst of Gaussian noise with the duration of 150 ms (including 30-ms rise-fall times), which was synthesized using the “randn()” function in the MATLAB function library (the Math Works Inc., Natick, MA, USA) at the sampling rate of 16 kHz with 16 bit resolution. All the acoustic signals, calibrated by a sound-level meter (AUDit and System 824, Larson Davis, USA), were delivered from a notebook computer sound card (ATI SB450 AC97) and presented by headphones (HD 265 linear, SENNHEISER, Germany).

#### Testing procedures

The PPI testing began with a 3 min adaptation period of the presentation of broadband background noise (60 dB SPL). Then a two-session PPI testing was conducted. The prepulse (broadband white noise, 150 ms, 65 dB SPL) was presented from the two headphones with an inter-ear onset delay being either +3 ms (left leading) or −3 ms (right leading). Due to the precedence effect^20,21^, in each of the participants with normal hearing, a single fused prepulse image would be perceived at the left ear in some trials (when the left-ear sound led to the right-ear one) and at the right ear in other trials (when the right-ear sound led). In fact, before testing, either the prepulse or the noise masker was presented to participants binaurally, and each of the participants reported that only a single prepulse image or a single noise image was perceived at the leading ear.

During the testing, in addition to the prepulse, a broadband noise (0–10 kHz, 60 dB SPL) was continuously delivered from the two headphones as the masker. The inter-ear onset delay for the masker was +3 ms (the left ear was the leading ear and the fused noise-masker image was at the left ear) in one testing session and −3 ms (the right ear was the leading ear and the fused noise-masker image was at the right ear) in the other session. Thus, two types of perceived laterality relationships between the prepulse and masker were created: perceptual separation (when prepulse and masker had different leading ears) and perceptual co-location (when prepulse and masker shared the same leading ear).

In a testing trial with the presentation of both prepulse and startling stimuli, the startling white-noise burst (40 ms, 104 dB SPL) started either 60 or 120 ms after the offset of the prepulse. The next testing trial started about 20 s later (varying from 15 to 25 s).

In each of the 2 testing sessions, 6 types of trials were used: (1) 4 prepulse/masker co-location trials with the ISI of 60 ms; (2) 4 prepulse/masker co-location trials with the ISI of 120 ms, (3) 4 prepulse/masker separation trials with the ISI of 60 ms, (4) 4 prepulse/masker separation trials with the ISI of 120 ms, (5) 8 startling-pulse-alone trials, and (6) 8 prepulse-alone trials. All the 32 trials were presented in a pseudo-random order for each participant.

To maintain participants’ attention across trials throughout each of the sessions, participants were asked to report the total number of the weak-sound burst (the prepulse) at the end of each of the 2 sessions (in total 24 prepulse stimuli were presented in a session). By doing so, the listening to the prepulse stimulus became an active listening task^29^, and participants’ attention to the prepulse signal was modulated by the shift between the co-location condition and the separation condition.

#### Data analyses

Startle eyeblink responses were recorded as electromyographic activity. Each trial was visually inspected for spontaneous and voluntary blinks. Trials with ocular artifact exceeding ±100 μV were excluded from analyses (trial exclusion rate <5%). The peak values of the startle-eyeblink EMG were averaged among the startle-alone trails and used as the value of “amplitude to startling sound alone”. The magnitude of PPI was calculated with the following generally used formula:

$$\begin{array}{rcl}{\rm{PPI}} & = & ({\rm{amplitude}}\,{\rm{to}}\,{\rm{startling}}\,{\rm{sound}}\,{\rm{alone}}\,-\,{\rm{amplitude}}\,{\rm{to}}\,{\rm{startling}}\,{\rm{sound}}\,{\rm{preceded}}\,{\rm{by}}\,{\rm{prepulse}})\\  &  & /({\rm{amplitude}}\,{\rm{to}}\,{\rm{startling}}\,{\rm{sound}}\,{\rm{alone}})\end{array}$$

Repeated-measures analyses of variances (ANOVAs) followed by Bonferroni pairwise comparisons and paired t-tests were performed using SPSS 15.0 software. The null-hypothesis rejection level was set at 0.05.

### Experiment 2: Effects of Perceptual Separation on ERPs to the Prepulse and on PPI

#### Participants

Twenty adults (8 males and 12 females, mean age = 22.7 ± 8.1 years) participated in Experiment 2. They were recruited from Peking University and gave their written informed consent to participate in this study. All participants were right-handed native Chinese speakers with normal (audiometric thresholds < 25 dB HL between 250 and 8000 Hz) and bilaterally balanced hearing (interaural threshold differences at each of the frequencies did not exceed 10 dB). The participants were paid a modest stipend for their participation.

#### Apparatus and stimuli

The apparatus for scalp electroencephalogram (EEG) recordings have been described in detail elsewhere^29,36^. Briefly, acoustic signals were transferred using a Creative Extigy sound blaster and presented to participants by two tube ear inserts used for ERP recordings (Neuroscan, El Paso, TX, USA). ERP recordings were conducted in the sound-attenuating booth (EMI Shielded Audiometric Examination Acoustic Suite) that was equipped with a 64-channel NeuroScan SynAmps system (Compumedics Limited, Victoria, Australia). EEG signals were recorded by the NeuroScan system with a sample rate of 1000 Hz and the reference electrode located on the nose.

During recordings (bandpass: 0.05–40 Hz; sampling rate: 1000 Hz), all electrodes were referenced to the electrode located on the nose. Eye movements and eye blinks were recorded from electrodes located superiorly and inferiorly to the left eye and at the outer canthi of the two eyes. Ocular artifacts exceeding ±100 μV were automatically rejected before averaging. A recording period including 100 ms before (served as the baseline) and 500 ms after the prepulse onset was used for data analyses. Trials contaminated by excessive peak-to-peak deflection (±100 μV) at channels not adjacent to eyes were automatically removed before averaging. For each participant, ERPs were then averaged separately for each combination of electrode site and stimulation condition. The averaged ERPs evoked by the prepulse sound under either the co-location or separation condition were analyzed across participants.

#### Testing procedures

All the 20 participants participated in two testing stages. To obtain the behavioral testing results in the participants in the ERP-recording experiment, in stage 1 of Experiment 2, the prepulse stimulus and the noise masker were presented from each the two headphones, and the startling white-noise burst (40 ms, 104 dB SPL) started 120 ms after the offset of the prepulse. Without the prepulse-pulse delay of 60 ms, all other stimulation parameters and experimental procedures were the same as used in Experiment 1.

In stage 2 (with 2 sessions), EEG responses to the prepulse stimulus were recorded. Note that in stage 2 of Experiment 2, only the prepulse and the noise masker were presented (the startling sound was not presented). The purpose of stage 2 of Experiment 2 was to examine the effect of perceptual separation on ERPs to the prepulse stimulus, and to provide ERP-recording results for the analysis of the correlation between the perceptual separation-induced PPI enhancement and the perceptual separation-induced ERP change. Two recording sessions were conducted in stage 2. In session 1, both the noise masker (60 dB SPL) and the target sound (i.e., the prepulse stimulus as used in Experiment 1) were presented from the two headphones with the inter-ear onset delay being −3 ms (right leading). Thus, the masker and target were perceived co-located in session 1.

In session 2, the masker was presented from the two headphones with the inter-ear onset delay being −3 ms (right leading), while the target sound was presented with the inter-ear onset delay being +3 ms (left leading), resulting in the perceptual separation between the masker and target.

Each session contained 150 trials (with the duration of 2000 ms for each trial), including 120 trials presenting both the masker and the target, and 30 trials presenting the masker only. Each trial started with the masker, and then the target was presented within an 800–1000 ms temporal window after the masker onset. Participants were instructed to attend to sounds presented from the headphones and press a button after a trial if they had not heard the target after the offset of the masker. To limit eye movements, participants were also asked to watch a cross in the center of the monitor screen in front of them. The interval between trials was 2000 ms.

#### Data analyses

In stage 1, the magnitude of PPI was calculated as described in Experiment 1.

In stage 2, the latencies and voltages of the N1 (the largest negative potential 100–210 ms after the target onset) and P2 components (the largest positive potential 210–350 ms after the target onset) were measured. Particularly, the averaged responses at the Cz electrode were statistically analyzed. Mixed and within-subject repeated-measures ANOVAs followed by Bonferroni pairwise comparisons and paired t-tests were performed using SPSS 15.0 software. The null-hypothesis rejection level was set at 0.05.
